# Marcello Malpighi: the nervous system under a microscope

**DOI:** 10.1590/0004-282X-ANP-2020-0309

**Published:** 2021-05-08

**Authors:** Eliasz ENGELHARDT

**Affiliations:** 1 Universidade Federal do Rio de Janeiro, Instituto de Neurologia Deolindo Couto, Setor de Neurologia Cognitiva e do Comportamento, Rio de Janeiro RJ, Brazil. Universidade Federal do Rio de Janeiro Universidade Federal do Rio de Janeiro Instituto de Neurologia Deolindo Couto Setor de Neurologia Cognitiva e do Comportamento Rio de Janeiro RJ Brazil; 2 Universidade Federal do Rio de Janeiro, Instituto de Psiquiatria, Centro de Doenças de Alzheimer e Outras Desordens Mentais na Velhice, Rio de Janeiro RJ, Brazil. Universidade Federal do Rio de Janeiro Universidade Federal do Rio de Janeiro Instituto de Psiquiatria Centro de Doenças de Alzheimer e Outras Desordens Mentais na Velhice Rio de Janeiro RJ Brazil

**Keywords:** Microscopy, Nervous System, Cerebral Cortex, Microscopia, Sistema Nervoso, Córtex Cerebral

## Abstract

The longstanding study of gross anatomy experienced a considerable improvement with the advent of the microscope in the early 17^th^ century. The representative personality of this new era certainly was Marcello Malpighi, seen as “founder of microscopic anatomy”. He studied, with a rudimentary compound microscope, numerous tissues and organs of several classes of animals, as well as plants. He described, for the first time, the microscopic structure of the nervous system, identifying in the gray matter of its various levels minute elements he took as “glands”. It should be reminded that the concept of “cell” (and “nerve cell”) was unknown at his time. Many researchers followed, performing microscopic studies, but without better results, and Malpighi’s view was maintained until the beginning of the 19^th^ century, when new histological processing and staining techniques appeared, as well as improved microscopes.

## RESUMO

O estudo de longa data da anatomia macroscópica experimentou um incremento considerável com o advento do microscópio no início do século 17. A personalidade representativa dessa nova era foi, certamente, Marcello Malpighi, considerado “fundador da anatomia microscópica”. Ele estudou, com um microscópio composto rudimentar, numerosos tecidos e órgãos de diversas classes de animais, assim como plantas. Descreveu, pela primeira vez, a estrutura microscópica do sistema nervoso, identificando na substância cinzenta dos vários níveis elementos de minúsculas dimensões, que denominou “glândulas”. Deve-se lembrar que o conceito de “célula” (e de “célula nervosa”) era desconhecido naquele tempo. Muitos pesquisadores seguiram realizando estudos microscópicos, mas sem resultados melhores, e o entendimento de Malpighi foi mantido até o início do século 19, quando apareceram técnicas histológicas novas de processamento e de coloração, assim como microscópios mais aprimorados.

## INTRODUCTION

**Figure 1.**
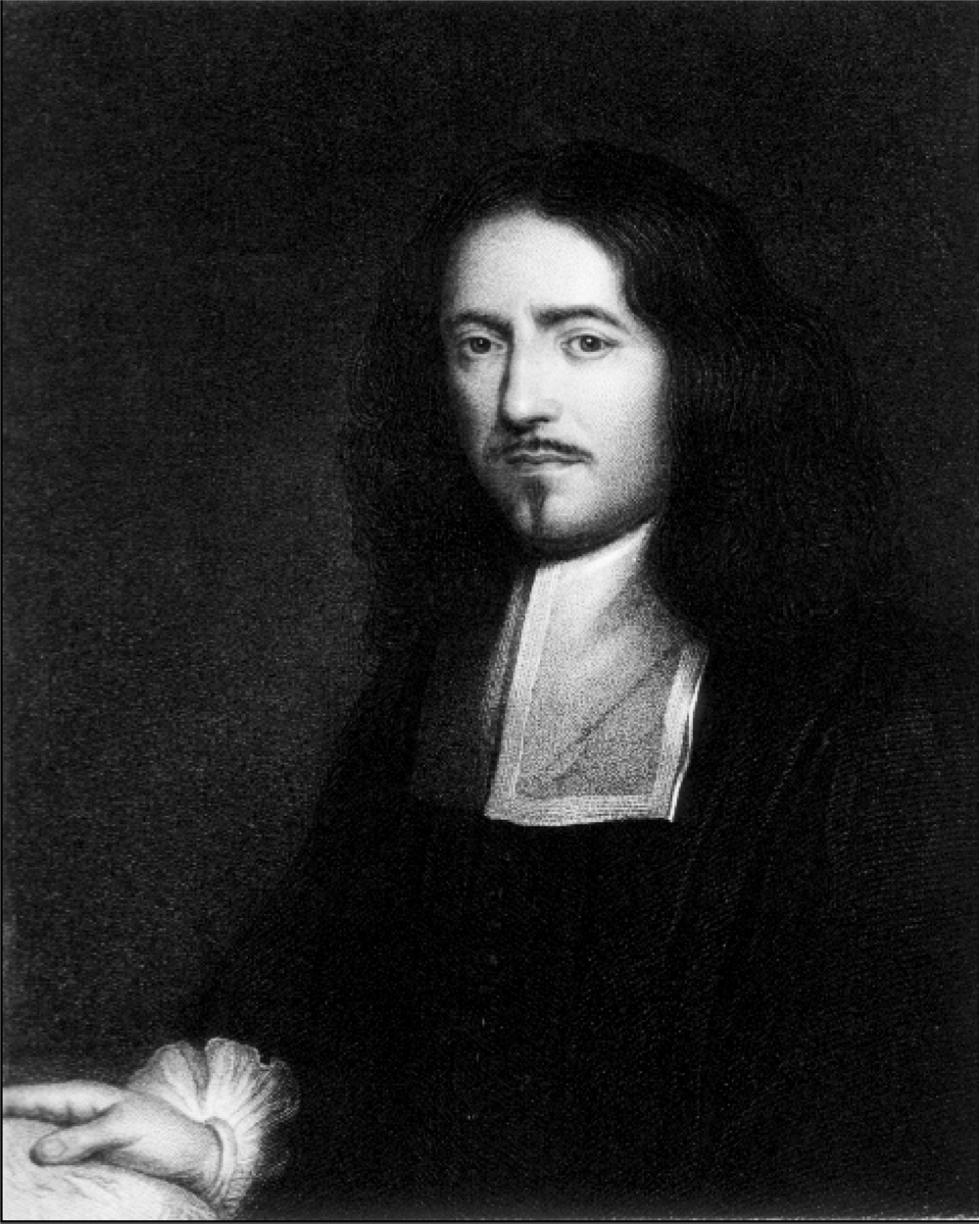
Marcello Malpighi (author LC Miall).

The study of anatomy has aroused the interest of innumerous researchers since the far antiquity, and gross features were revealed through dissection of varied classes of animals, and also, in some periods, of human corpses^1^^,^^2^. The advent of the microscope permitted to expand the anatomic findings with the study of the fine structure of tissues and organs^3^. Among the researchers who pioneered such studies, the name of the Italian physician, biologist, botanist, and anatomist Marcello Malpighi (1628-1694), later seen as the “founder of microscopic anatomy” (histology), must be stressed^4^ (Figure 1).

Public domain. Available at: https://commons.wikimedia.org/wiki/File:MarcelloMalphigiMiall.jpg

## MALPIGHI’S CAREER

Malpighi obtained the degree of Philosophy and of Medicine (1653), and became successively Professor of Medicine at the Universities of Bologna, Pisa, and Messina. He was introduced to the microscope while in Pisa (1656), being apparently one of the first to use the compound instrument (objective plus ocular lenses), turning possible much of his research. He moved to Rome after accepting the invitation to be Pope Innocent XII personal physician (1691)^4^^,^^5^^,^^6^, where he also taught Medicine in the Papal Medical School, and continued treating patients, in person and by post^6^^,^^7^. He authored many important scientific contributions on the structure of animals, describing varied tissues and organs, as the liver, brain, lung and pulmonary circulation (with the discovery of the capillaries), heart, kidney, spleen, skin, tongue, and many of his findings would later carry his name. Additionally, he studied the chick embryo development, insects, and plants^8^. His microscopic findings on the nervous system were novel and important, and here some aspects will be highlighted.

## THE NERVOUS SYSTEM

Malpighi’s investigations on the nervous system were first published in *De Cerebro Epistola* (1665)^9^. Next, he presented in *De cerebri cortice* (1666) a more detailed description of the microscopic structure of the nervous system, with emphasis on the “cortex of the cerebrum” (*cerebri cortice*) [cerebral cortex], apparently for the first time^10^^,^^11^^,^^12^. The studies were performed through sections of material of various classes of vertebrates, and the findings resulted from dissection, and microscopic examination^9^^,^^10^.

His initial account was on the general anatomic aspects of the nervous system of “perfect animals” (higher animals [mammals]), as known at the time^9^^,^^10^^,^^11^. However, his main target was to know the minute composition of these structures “…to acquire a better knowledge of the smallest structures of this substance [nervous tissue] …up to now unclear…”^10^^,^^12^^,^^13^. He reported that the cerebral cortex was constituted by a collection of tiny “glands” (“cells” -“nerve cells”) with an oval shape, some forming obtuse angles when compressed by adjacent elements (glands). They were connected to roots of white nervous fibers, like special vessels, analogous to the excretory canals of true glands. The cortical substance (gray matter - glands) was also found inside the cerebral ventricles (basal nuclei), in the beginning of the spinal medulla (medulla oblongata) (brain stem), in the external sheet of the cerebellum, scattered in many places under the pons of Varolius, and inside the entire spinal medulla (spinal cord). He described also the already better-known fibers that formed the white matter (1666)^10^^,^^12^^,^^13^^,^^14^ (Table 1).

**Table 1.** Excerpts of Malpighi’s microscopic description of the nervous system^10^^,^^12^^,^^13^^,^^14^.

The studies were performed through sections of material of sanguineous perfect animals [*perfectorum animalium*] (higher animals [probably mammals]), larger fishes, and other classes of vertebrates. The findings resulted from dissections of cooked material, eventual pouring of ink and then lightly wiping away, and microscopic examination. Regarding the [cerebral] gray matter, he reported: “…in the brain of perfect animals the cerebral cortex is formed by a collection of tiny glands [nerve cells], found in the cerebral gyri…”. And: “They have an oval shape, but, as they are compressed by adjacent [glands], some form obtuse angles…”. Then: “Their external portion is in contact with the pia mater and its blood vessels, which penetrate from above into their substance…the interior part gives off white nervous fibers, like special vessels, analogous to the excretory canals of true glands…”. And more: “…the medullary matter [white matter] is a result of the assemblage of many kinds of small [nervous] fibers jointed together…”. Further: “These cortical glands…make up the external cerebral gyri and are connected with the medullary fibers which take their origin from them, in such a way that, wherever gyri are cut transversely, a fixed and constant mass of glands is surrounding the white matter…observed more clearly in the cerebellum…” Describing deeper structures: “…the cortical substance [gray matter] inside the cerebral ventricles [prominences of the ventricles] [basal nuclei] is of the same nature as that of outside of the brain [i.e., glands], and the same is also seen in the beginning of the spinal medulla…”. And: “Inside of the entire spinal medulla [spinal cord] the cortical matter [gray matter] already described maintain the glandular structure, and…under the pons of Varolius… such cortical glands are scattered in many places…”. Further: “This also happens in the cerebellum, where the nerves leaving the pons of Varolius, and entering inside the cerebellum, have also their origin in glands of the cortical matter found there…”. About fiber tracts: “The spinal medulla is a fascicle of nerves that on attaining the cerebrum is divided into two parts, turning smoothly to form the lateral aspects of the ventricles...they end in the cortical matter where the extremities of the roots are inserted in the small grains of its glands…”. And: “…in the cerebrum and cerebellum, white fibers are seen by which the white matter is formed…they serve as vehicles of the nervous juice (*succus nervosus*)…an intimate connection and continuation between these cortical glands and nervous fibers is observed...”.

**Figure 2.**
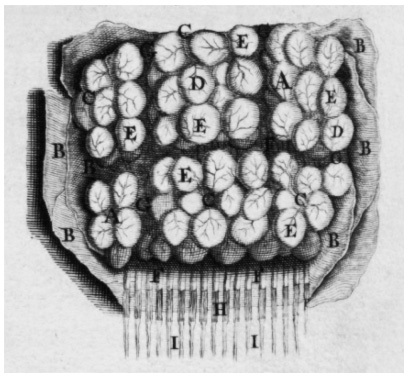
Drawing of the cerebral cortex (From Bidloo G. *Anatomia humani corporis*, 1685 (Table X, Figure 2 from Bidloo's book)^15^ as described by Malpighi^12^^,^^13^.

There is no illustration of the described nervous system in his *De cerebri cortice*. A drawing of the cortex, according to Malpighi’s description, may be found only in Govard Bidloo’s *Anatomia humani corporis* (plates drawn by Gérard de Lairesse) (1685) (Table X, Figure 2 from Bidloo's book)^13^^,^^15^ (Figure 2).

A: part of boiled brain; B: meninges wrapping the brain; C: Blood vessels; D: their capillary distribution & reticular plexus; E: various rows of cortical glands; F: tubules (nerve fibers or vessels) assembled in fasciculi, constituting the medulla [white matter]; G: lobular fascicles, or separations; H: complicated tubules (vessels) (nerve fibrils); I: nerves. [F+H+I= white roots of nerves]^13^^,^^15^.

## COMMENTS

Malpighi provided the first microscopic description of the central nervous system of “perfect animals" (superior animals [probably mammals]). He focused on the gray matter, defined as “cortical”, in the various levels of the nervous system. There he identified microscopic discrete elements he denominated “glands” in the gray matter of the cerebral and cerebellar cortex, basal nuclei, brain stem, and spinal cord. The fibrous structures, he called “nerve fibers” (or vessels, or excretory ducts), were constituents of the already known subcortical white matter and tracts. His interpretation of the cortical elements as “glands” may be related to his admiration for Hippocrates, who regarded the brain as a glandular organ (*Cerebrum inter glandulas recenset*, as he wrote, based on *De GIandulis*), to a homology to other organs he identified as glandular (e.g., liver)^12^, and supporting a presumed glandular function of the cortical matter (cerebral and cerebellar cortex), responsible for filtering the blood to produce “nervous juice” (animal spirit), carried by nerve fibers (vessels) [ducts] coursing downward (to the medulla oblongata and spinal cord) (in accordance to Thomas Willis view)^12^^,^^13^^,^^16^. It is important to highlight that at the time, although the term “cell” was already known, due to Robert Hooke’s studies (1665), the concept of “cell” was introduced much later, by Theodor Schwann and Matthias Schleiden (1839), while the concept of “nerve cell” was yet unknown, beginning to appear only in the second half of the 19^th^ century, mainly thanks to Jan Evangelista Purkinje, describing “ganglionic bodies” in the gray matter of the brain (1837)^3^^,^^17^.

The microscopic findings of the cortical matter (the glands) generated adverse arguments, frequent at his time, and more recently some authors claimed, mainly in relation to the nervous system, that they represented artefacts^4^^,^^13^^,^^18^. Others accepted that they represented valid structures^10^^,^^13^^,^^18^. It should be considered that the recent criticism was mostly directed to the drawing of the cerebral cortex, and less to the original description. It must be remembered that the real author of this depiction remained undefined, and that probably it was drawn based on Malpighi’s description, and not from direct observation of the specimen under the microscope^13^. Curiously, such debate was only directed to the findings of the cerebral cortex, and not to the other levels, neither to the observations of other tissues and organs, studied with the same precarious methods, which were, on the contrary, praised^13^.

It can be affirmed, without error, that Malpighi’s findings, identifying minute elements in the gray matter of the nervous system, an unshaped tissue until his time, was a large new step to elucidate its structure. Following Malpighi, many researchers performed microscopic examination of the nervous system. However, all their reports looked unremarkable compared to Malpighi's description. His view and the representation of the cerebral cortex were afterwards reproduced by many authors, until the beginning of the 19^th^ century, when new histological processing and staining techniques appeared, as well as improved microscopes^3^^,^^13^.
